# Data on the correlation between hemostatic parameters before treatment start, and markers of fibrinolysis during treatment in patients with acute pulmonary embolism, undergoing ultrasound-assisted catheter-directed thrombolysis

**DOI:** 10.1016/j.dib.2025.112131

**Published:** 2025-10-11

**Authors:** Dominik F. Draxler, Justine Brodard, Heidi Ho, Konstantina Chalkou, Charithani B. Keragala, Thomas Lillicrap, Dik Heg, Johanna A. Kremer Hovinga, Robert L. Medcalf, Anne Angelillo-Scherrer, Stefan Stortecky

**Affiliations:** a Department of Cardiology, Inselspital, Bern University Hospital, University of Bern, Freiburgstrasse 20, CH-3010 Bern, Switzerland; b Bern Center of Precision Medicine, Department of Biomedical Research, University of Bern, Murtenstrasse 28, CH-3008 Bern, Switzerland; c Department of Hematology and Central Hematology Laboratory, Inselspital, Bern University Hospital, University of Bern, Freiburgstrasse 18, CH-3010 Bern, Switzerland; d Molecular Neurotrauma and Haemostasis, Australian Centre for Blood Diseases, Monash University Melbourne, 99 Commercial Road, Melbourne VIC 3004, Australia; e Department of Clinical Research, University of Bern, Freiburgstrasse 3, 3000 Bern, Switzerland; f Department of Neurology, John Hunter Hospital, 156 Kookaburra Cct, New Lambton Heights NSW 2305, Newcsatle, Australia; g Hunter Medical Research Institute, University of Newcastle, 156 Kookaburra Cct, New Lambton Heights NSW 2305, Newcsatle, Australia

**Keywords:** Pulmonary embolism, Catheter-directed thrombolysis, Fibrinolysis, Fibrinolytic potential, Fibrinolytic capacity

## Abstract

Personalized medicine is nowadays increasingly being used to tailor therapies to the individual needs of patients [1]. We recently demonstrated, that various fibrinolytic parameters, including the plasma-based inducible plasmin-antiplasmin (plap) complex, as well as viscoelastic parameters, predict the individual treatment response in patients with intermediate- or high-risk pulmonary embolism (PE), treated with ultrasound-assisted catheter-directed thrombolysis (USAT) [2]. Markers of fibrinolysis and coagulation were assessed before the start of treatment (t0) and during the 10 - 15-hour infusion of recombinant tissue-type plasminogen activator (rt-PA) (t6). While we have already provided evidence of the predictive value of the assessed parameters for efficacy outcomes, in this article we present data on the correlation between these predictive markers and the endogenous fibrinolytic response during USAT. These data confirm the predictive value of the identified pre-treatment parameters for the endogenous treatment response, which may be important for their potential usefulness in modulating treatment decisions and dose adjustments in USAT for PE in the future.

Specifications Table

**Table:** 

Subject	Health Sciences, Medical Sciences & Pharmacology
Specific subject area	Cardiology, Fibrinolysis
Type of data	Tables, analysed
Data collection	In a single-center cohort study of USAT for intermediate-high or high-risk PE, plasma samples were collected from 41 patients before treatment start and at 6 h (during infusion of rt-PA), as part of the BERn Acute Pulmonary EmboliSm REgistry (ERASE PE).
Data source location	Institution: University hospital of Bern, City/Town/Region: Bern, Switzerland Country: Switzerland
Data accessibility	Repository name: “Data on the correlation between hemostatic parameters before treatment start, and markers of fibrinolysis during treatment in patients with acute pulmonary embolism, undergoing ultrasound-assisted catheter-directed thrombolysis”; Mendeley Data, V2 Data identification number: 10.17632/czyy7jbnb7.2 Direct URL to data: https://data.mendeley.com/datasets/czyy7jbnb7/2
Related research article	“The Individual Fibrinolytic Capacity Predicts Efficacy Of Ultrasound-Assisted Catheter-Directed Thrombolysis In Patients With Acute Pulmonary Embolism” Draxler et al. Journal of Thrombosis and Haemostasis 2024 doi:10.1016/j.jtha.2024.12.043.

## 1 Value of the data

• The individual fibrinolytic potential has previously been shown to predict the treatment response in patients with PE, treated with USAT, based on well-recognized efficacy outcomes. These data demonstrate that various identified predictive parameters also correlate well with the extent of the endogenous fibrinolytic response.
• Several fibrinolytic parameters, including the novel inducible plap complex assay, assessed *ex vivo* before treatment start, correlate with endogenous plasmin generation and activation of fibrinolysis.
• Viscoelastic testing is a well-established diagnostic tool in various clinical settings. These data demonstrate, that pre-treatment viscoelastic parameters also correlate with the endogenous capacity to activate plasmin generation and fibrinolysis.
• Personalised treatment approaches may be harnessed in the future to guide treatment decisions and dosing regimens in intermediate- to high-risk PE. These new data support the ability of pre-treatment fibrinolysis and coagulation parameters to reflect the individual endogenous response.

## 2 Background

Acute PE is a common cardiovascular emergency with an incidence of 1 in 1000 annually and early mortality up to 30 % [[1], [2], [3], [4]]. Intermediate-high and high-risk PE patients with right ventricular overload often require urgent reperfusion via intravenous thrombolysis using plasminogen activators like rt-PA [5,6]. However, systemic thrombolysis carries a bleeding risk in up to 20 % of patients, including 2–5 % risk of intracranial hemorrhage [7]. Catheter-directed thrombolysis, particularly USAT, has emerged as a lower-dose alternative [8].

Personalized medicine aims to adapt medical therapies to the specific requirements of individual patients [9]. We recently showed that several fibrinolytic parameters, including the plasma-based inducible plasmin–antiplasmin (plap) complex and viscoelastic measurements, can predict individual treatment responsiveness in patients with intermediate- or high-risk pulmonary embolism (PE) undergoing ultrasound-assisted catheter-directed thrombolysis (USAT) [10]. Markers of fibrinolysis and coagulation were evaluated both before treatment initiation (t0) and during the 10 to 15-hour infusion of recombinant tissue-type plasminogen activator (rt-PA) (t6). While previous work has demonstrated the predictive value of these parameters for treatment efficacy, this article focuses on their relationship with the endogenous fibrinolytic response during USAT. Our findings further support the utility of these pre-treatment markers in predicting the individual response to therapy, which could be crucial for guiding treatment strategies and dose adjustments in USAT for PE moving forward.

## 3 Data Description

All raw and analysed data are available in the Mendeley Data Repository “Data on the correlation between hemostatic parameters before treatment start, and markers of fibrinolysis during treatment in patients with acute pulmonary embolism, undergoing ultrasound-assisted catheter-directed thrombolysis” [11]. Markers analysed can be characterized as follows:

**Markers of fibrinolysis assessed before treatment start:**

t0_plasminogen
t0_α2-antiplasmin
t0_plap complex
Baseline plap complex
t-PA – induced plap complex / baseline
t-PA – induced plap complex (with co-factor) / baseline
fibrin sensitivity ratio
t0_u-PA antigen
t0_u-PA activity t0_D-dimer

**Viscoelastic testing before treatment start:**

t0_(CT or CFT or A10 or α-angle or MCF or ML) EXTEM t0_(CT or CFT or A10 or α-angle or MCF or ML) INTEM t0_(CT or CFT or A10 or α-angle or MCF) FIBTEM
t0_(CT or CFT or A10 or α-angle or MCF) HEPTEM

**Global coagulations tests and fibrinogen levels before treatment start**

t0_INR
t0_Quick
t0_aPTT
t0_fibrinogen

**Markers reflecting plasmin generation and fibrinolytic activity during thrombolytic treatment:**

t6_plasminogen
t6_α2-antiplasmin
t6_plap complex
t6_u-PA antigen
t6_u-PA activity t6_D-dimer
t6_ML EXTEM
t6_ML INTEM

**Markers of changes in coagulation and fibrinogen consumption during thrombolytic treatment:**

t6_CT (EXTEMN or INTEM or FIBTEM or HEPTEM)
t6_CFT (EXTEMN or INTEM or FIBTEM or HEPTEM)
t6_A10 (EXTEMN or INTEM or FIBTEM or HEPTEM)
t6_α-angle (EXTEMN or INTEM or FIBTEM or HEPTEM)
t6_MCF (EXTEMN or INTEM or FIBTEM or HEPTEM)
t6_INR
t6_Quick
t6_aPTT
t6_fibrinogen

## 4 Experimental Design, Materials and Methods

### 4.1 Global coagulation tests, fibrinogen and **d**-dimer levels

Global coagulation tests, including international normalized ratio (INR), Quick activated partial thromboplastin time (aPTT), fibrinogen levels and d-dimer were assessed in citrated plasma by the central clinical hematology laboratory of our hospital, harnessing established protocols used in clinical routine. Global coagulation tests were performed using coagulometry. d-dimer levels were evaluated by enzyme-linked immunosorbent assay (ELISA). Fibrinogen levels were also assessed by coagulometry (Clauss).

### 4.2 Assessment of plasminogen activity

Plasminogen activity was evaluated by the central clinical hematology laboratory at Inselspital Bern using their established protocol. Briefly, plasma samples were exposed to an excess of streptokinase, which forms an active plasminogen-streptokinase complex. To maintain consistent fibrinogen levels and avoid falsely elevated plasminogen readings in samples with increased fibrinogen or fibrin(ogen) breakdown products, the streptokinase reagent contains plasminogen-poor fibrinogen. The activity of the plasminogen-streptokinase/fibrinogen complex was measured by monitoring the hydrolysis rate of the chromogenic substrate SPm41. A standard human plasma sample with a defined plasminogen activity was used as a reference, and plasminogen activity was reported as a percentage relative to normal plasma.

### 4.3 Evaluation of α2-antiplasmin activity

The hospital’s central clinical hematology laboratory assessed α2-antiplasmin activity following their standard procedure. In summary, plasma samples were exposed to an excess of plasmin. The α2-antiplasmin present in the sample binds and neutralizes an equivalent amount of plasmin by forming plasmin–α2-antiplasmin complexes. The remaining plasmin was measured by its ability to cleave a chromogenic substrate added to the mixture. The α2-antiplasmin activity was then determined using a calibration curve and expressed as a percentage of normal plasma, referencing standard human plasma with a manufacturer-specified α2-antiplasmin activity.

### 4.4 Assessment of plasma urokinase-type plasminogen activator (u-PA) levels

The u-PA plasma concentrations were determined using the TECHNOZYM u-PA ELISA Kit (Technoclone, catalogue number TC12010) according to the manufacturer’s protocol. Wells coated with monoclonal anti-u-PA antibody were washed and then incubated with a conjugated polyclonal anti-u-PA antibody for one hour at 37 °C. After further washing, substrate solution was added and incubated at room temperature for 20 min before stopping the reaction with a stop solution. Absorbance readings were taken at 450 nm.

### 4.5 Evaluation of plap complex levels

Plasma levels of plasmin–α2-antiplasmin (plap) complexes were assessed using the DRG PAP ELISA RUO Kit (DRG Instruments, Marburg, Germany, catalogue number EIA-3763) following the manufacturer’s guidelines.

### 4.6 *Ex vivo* inducible plap complex levels

Changes in plap complex levels before and after addition of t-PA were used as a surrogate measure for baseline and inducible plasmin generation, respectively. For inducible plap complex formation, plasma (40 µL) was treated ex vivo with 50 nM t-PA for 5 min in a 96-well polymerase chain reaction plate to generate plasmin in the presence or absence of the fibrin-like co-factor cyanogen bromide activated fibrinogen (CNBr-fibrinogen, 0.2mg/ml, manuscript in preparation). This was done in a Bio-Rad Thermal Cycler preset to 37 °C. After treatment, 20 µL of each sample was transferred using an 8-channel pipette to a second 96-well plate (on ice) containing 180 µL of 21.1 µM of aprotinin (final concentration, 20 µM) to stop further plap complex formation. The aprotinin-blocked samples were assessed for plap complex levels using the DRG PAP ELISA RUO Kit (DRG Instruments, Marburg, Germany, catalogue number EIA-3763). We further calculated the ratio between plap complex levels generated with and without CNBr-fibrinogen, resulting in the fibrin-sensitivity ratio.

### 4.7 Viscoelastic testing

Viscoelastic whole blood testing was carried out using Rotational Thromboelastometry (ROTEM®, Werfen France). Four assays were performed to assess coagulation status before (pre-lysis) and during (6 h after start) USAT treatment. The EXTEM assay activates the extrinsic coagulation pathway through tissue factor, while the INTEM assay triggers the intrinsic pathway using negatively charged agents like kaolin or ellagic acid. The FIBTEM assay evaluates fibrinogen activity by adding cytochalasin, which inhibits platelet microfilaments and thus eliminates the platelet contribution to clot retraction. Because all patients were treated with heparin, the HEPTEM assay was also utilized, where heparinase neutralizes heparin’s anticoagulant effect. The analyzed parameters included clotting time (CT, seconds), clot formation time (CFT, seconds), α-angle (degrees) indicating fibrin polymerization rate, clot firmness at 10 min (A10, mm), maximum clot firmness (MCF, mm), and maximum clot lysis (ML, %) to assess fibrinolysis [12].

## Limitations

Although some of the identified predictive markers correlate well with the endogenous treatment responsiveness in PE patients undergoing USAT, it is important to emphasize that this data set was derived from a pilot trial and is therefore underpowered to support definitive conclusions. Moreover, this investigation was performed as a single-center study, hence generalisability is not guaranteed. It is also worth noting that the assays and parameters employed—while approved in other contexts (e.g., viscoelastic testing)—are not yet validated or standardized for this specific indication.

## Ethics Statement

Consecutive patients with PE referred for advanced PE care were considered for study inclusion. After arrival at the tertiary care center, patients were evaluated based on clinical presentation, hemodynamics, and imaging details by an experienced interdisciplinary PE response team. Risk stratification, decision-making, and the treatment of PE using advanced modalities are performed as a 24/7 service at the Center for PE under the lead of the Department of Cardiology. The study protocol received approval from the local ethics committee, and all patients gave written informed consent prior to participation in the investigation.

## Credit Author Statement

**D.F.D, J.B, A.A.-S, R.L.M. and S.S:** Conceptualization, Methodology. **D.F.D:** Data curation, Writing, Original draft preparation. **J.B, H.H, C.B.K, R.L.M, T.L, D.H. and J.K.H:** Investigation and statistical analyses. All authors: Reviewing and Editing.

## Data Availability

Mendeley DataData on the correlation between hemostatic parameters before treatment start, and markers of fibrinolysis during treatment in patients with acute pulmonary embolism, undergoing ultrasound-assisted cat (Original data)
